# Risk of Urinary Recatheterization for Thoracic Surgical Patients with Epidural Anesthesia

**DOI:** 10.26502/jsr.10020068

**Published:** 2020-06-22

**Authors:** Luis E. De León, Namrata Patil, Philip M. Hartigan, Abby White, Carlos E. Bravo-Iñiguez, Sam Fox, Jeffrey Tarascio, Scott J. Swanson, Raphael Bueno, Michael T. Jaklitsch

**Affiliations:** a Division of Thoracic Surgery, Brigham and Women’s Hospital, Harvard Medical School, Boston, Massachusetts, USA; b Department of Anesthesiology, Perioperative and Pain Medicine, Brigham and Women’s Hospital, Harvard Medical School, Boston, Massachusetts, USA

**Keywords:** Thoracic Surgery, Epidural Anesthesia, Urinary Retention, Urinary Recatheterization, Benign Prostatic Hyperplasia

## Abstract

**Background:**

Current quality guidelines recommend the removal of urinary catheters on or before postoperative day two, to prevent catheter-associated urinary tract infections (CAUTI). The goal of this study was to evaluate the impact urinary catheter removal on the need for urinary recatheterization (UR) of patients with epidural anesthesia undergoing thoracic surgery.

**Materials and Methods:**

All patients undergoing thoracic surgery between November 4^th^, 2017 and January 9^th^, 2018 who had a urinary catheter placed at the time of intervention were prospectively evaluated. Patient characteristics including: history of benign prostatic hyperplasia (BPH), catheter related variables and rates of UR were collected through chart review and daily visits to the wards. BPH was defined as history of transurethral resection of the prostate or treatment with selective α_1_-adrenergic receptor antagonists.

**Results:**

Over a two-month period 267 patients were included, 124 (46%) were male. Epidural catheters were placed in 88 (33%) patients. Median duration of urinary catheters for the cohort was 1 day (0 days – 18 days), and it was significantly higher in patients with epidural anesthesia (Table 1). Overall 20 (7%) patients required UR. On initial analysis, there was no statistical difference in the rate of UR among patients with and without epidural catheters [9/88 (10%) vs 11/179 (6%), p=0.23). The rate of UR was higher in males than in females (14/124 (11%) vs 6/143 (4%), p=0.03). Fifteen (12%) patients had a diagnosis of BPH. The rate of UR was three-times higher in this group than in those without BPH [4/15 (27%) vs 10/109 (9%) p=0.05]. Four (1%) patients developed a CAUTI during follow-up, and the rate of CAUTI was not different between those with and without epidural catheters.

**Conclusion:**

Urinary catheters in patients with thoracic epidural anesthesia can be safely removed, as evidenced by low reinsertion and infection rates. Removal of urinary catheters in patients with a history of BPH should be carefully evaluated, as over 1/4 will require urinary recatheterization in this subgroup. Further study of this group is needed to avoid unnecessary patient discomfort associated with recatheterization.

## Introduction

Thoracic epidural anesthesia is commonly used in the perioperative pain management of patients undergoing major thoracic surgery, especially since it has shown to have a low incidence of postoperative morbidity [1]. One of the most common side-effects of an epidural is postoperative urinary retention, with incidence rates ranging from 5% to 26% [2]. The mechanism behind this is thought to be an impaired contractile response to muscarinic stimulation due to blocked transmission of afferent and efferent nervous impulses from and to the bladder [3].

Since patients often cannot sense bladder distention with an epidural, it has become common practice to leave urinary catheters until after the epidural has been discontinued to avoid urinary retention and the need for urinary recatheterization (frequently a painful procedure). Several studies, however, have demonstrated that prolonged urinary catheterization is associated with higher incidences of catheter associated urinary tract infections (CAUTI) [4, 5].

In 2005, the Surgical Care Improvement Project (SCIP) was introduced with the goal of improving patient care by reducing surgical complications [6]. In 2009 the SCIP Inf-9 was added to these guidelines, recommending the removal of all urinary catheters on or before postoperative day 2, to avoid CAUTI. However, the impact of this practice on patients undergoing thoracic surgery with epidural catheters remains unclear. Early removal of urinary catheters in patients with thoracic epidural anesthesia may decrease the incidence of CAUTI’s but may also result in increased urinary retention and need for recatheterization [7].

The goal of this study was to prospectively evaluate the impact of urinary catheter removal on the incidence of urinary tract infections and the need for urinary recatheterization of patients with epidural pain control undergoing thoracic surgery at a single large-volume institution.

## Materials and Methods

All patients who underwent thoracic surgery at Brigham and Women’s Hospital between November 4^th^, 2017 and January 9^th^, 2018, and had a urinary catheter placed at the time of surgery, were prospectively included. Diagnoses were grouped into six categories: pulmonary resections (wedge resection, segmentectomy, lobectomy, pneumonectomy); esophageal procedures (esophagectomy, myotomy, fundoplication, bronchopleural fistula closure, esophageal perforation repair); pleural procedures (pleurectomy, decortication, pleurodesis); mediastinal resections (thymectomy, mediastinal mass resection); chest wall procedures (rib resection, chest wall mass resections, chest wall reconstruction); and hernia repairs. Lung transplant patients were not included. Medical records for all cases were reviewed to verify the eligibility of patients for inclusion. This study was approved by the Institutional Review Board at Harvard Medical School and informed consent was waived.

All patients underwent placement of a urinary catheter in the operating room using standard sterile technique after induction of general anesthesia. Patients were divided into two groups, based on whether they had an epidural catheter placed preoperatively or not. All epidural catheters were placed by qualified anesthesiologists in the preoperative unit. Standard practice was to insert epidural catheters preoperatively at thoracic vertebral interspace number 6–7 (+/− 1 level), advance multiport catheters 5 cm into the epidural space, test-dose with 3 mL 2% lidocaine with epinephrine (1:200,000), and to initiate blockade during the last hour of the case with boluses of 10–15mL bupivacaine (0.125%) followed by a continuous infusion of same at 6mL/hr.

In general, epidural catheters were removed once pain was controlled adequately with oral and/or parenteral analgesia, usually within 4 days postoperatively. Bladder scans were performed, if patients had not voided for 8 hours after urinary catheter removal or complained of urinary fullness or pain. Urinary catheter reinsertion was performed if bladder scan showed a volume >200cc.

Data were collected prospectively by the thoracic surgery team during the postoperative course. Daily visits to the hospital wards were performed to assess the duration of epidural and urinary catheters and the need for recatheterization. When indicated, results of bladder scans, urinalyses, and urine cultures were obtained from radiology and clinical pathology reports by daily chart review.

A stratified analysis for males was conducted, to assess the effect of benign prostatic hyperplasia (BPH) on the need for urinary recatheterization. The diagnosis of BPH was determined from the history and physical note at the time of admission. Patients were considered to have BPH if they had a history of transurethral resection of the prostate (TURP) or were being treated with selective α_1_-adrenergic receptor antagonists (Tamsulosin, Terazosin and Doxazosin). Patients who required recatheterization prior to admission were not included.

Urinary tract infection was defined as the presence of genitourinary symptoms (suprapubic tenderness, dysuria, frequent / urgent need to urinate) in the setting of inflammation demonstrated by leukocytosis and/or a positive urinalysis. A urinalysis was considered positive if there were >5 white blood cells per high-powered field, or it was positive for leukocyte esterase and/or nitrites. A urine culture was ordered for patients with a positive urinalysis. The diagnosis of CAUTI was only made if there was a documented microbiologic pathogen on the urine culture.

### Statistical Analysis

Descriptive statistics for categorical variables are expressed as frequency and percentages; continuous variables are expressed as mean and standard deviations or median and ranges, as appropriate. Two-tailed Fisher exact test or chi-square were used to compare categorical variables. as appropriate. Mann-U Whitney tests were used for continuous variables. Statistical analyses were performed using SPSS 24 (IBM Corp, Armonk, NY). A p-value ≤0.05 was considered statistically significant.

## Results

Of the 267 patients, 124 (46%) were male and 143 (54%) were female. Median age at surgery was 66 years, range (17 years – 88 years) (Table 1). Operations included pulmonary resections in 135 (51%), esophageal procedures in 43 (16%), major pleural procedures (pleurectomy and decortication) in 39 (15%), mediastinal resections in 19 (7%), chest wall procedures in 19 (7%), and hernia repairs in 12 (4%). The number of patients undergoing esophageal and pleural procedures was higher in the epidural group, while the number of patients undergoing pulmonary resections was higher in the non-epidural group (Table 1).

Epidural catheters were placed in 88 (33%) patients. Median duration of epidural catheters was 4 days, range (1 – 9 days). Median duration of urinary catheters for the cohort was 1 day, range (0 days – 18 days), and it was significantly higher in patients with epidural analgesia (Table 1). Median hospital length of stay was 4 days (0 days – 3 months), and it was significantly longer in patients with epidural catheters (Table 1).

Overall 20 (7%) patients required urinary recatheterization. Urinary catheters were reinserted in 9 (10%) patients with epidural catheters, compared with 11(6%) patients who did not have an epidural in place. There was no statistical difference between groups at this level of analysis (Table 1). Gender, however, was a significant factor on the need for urinary recatheterization, with 14 (11%) males requiring reinsertion, compared with only 6 (4%) females (p=0.03).

**Figure 1:**
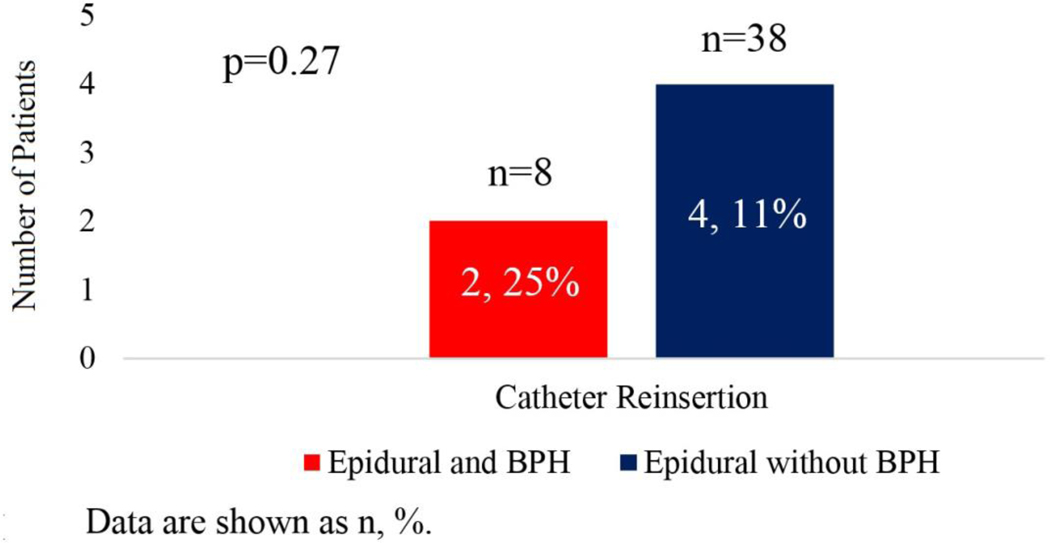
Catheter reinsertion in male patients with epidural catheters and benign prostatic hyperplasia (BPH) status.

Among the 124 males in the cohort, 15 (12%) patients met our criteria of BPH. All of them were on medication (Flomax), and two had a history of TURP. When comparing the rate of urinary recatheterization between males with and without BPH, the rate of urinary retention requiring recatheterization was three-times higher in patients diagnosed with BPH (Table 2). Looking only at patients with epidural catheters, 25% of those with benign prostatic hyperplasia required recatheterization, compared to 11% of those without BPH (Figure 1).

Four patients out of 267 (1%) had a documented urinary tract infection, three of which occurred in female patients, and 3/88 (3%) with epidural catheters had a UTI. There was no statistically significant difference between groups (Table 1). None of the patients who developed a UTI required urinary recatheterization.

## Discussion

While the potentially harmful effects of bladder overdistention argue in favor of postoperative indwelling catheterization for the duration of epidural analgesia, the risk of urinary tract infections associated with prolonged catheterization makes a case for early catheter removal.

Although there remains uncertainty regarding the optimal timing of urinary catheter removal in thoracic surgical patients with thoracic epidural analgesia, it is our hypothesis that early removal of urinary catheters is beneficial in reducing the risk of infection, as well as improving patient comfort. However, this must be balanced against urinary retention that can lead to the discomfort of catheter reinsertion and the complications of re-catheterization, which include urethral trauma and hematuria [8, 9].

Current literature presents conflicting evidence on the topic. In a randomized control trial, Zaouter et. al [10] evaluated 215 patients undergoing thoracic or abdominal procedures with thoracic epidural analgesia, randomizing patients to urinary catheter removal on postoperative day 1 versus removal after discontinuation of the epidural catheter. In this study, the rate of re-catheterization was 7.6% and it was higher in the early removal group, however, it did not reach statistical significance. The rate of urinary tract infections was significantly lower in the early catheter removal group. Based on these findings, this group recommended early removal of urinary catheters in this patient population. On the other hand, Allen et al. [7] randomized 247 patients undergoing thoracic procedures with thoracic epidural analgesia to urinary catheter removal within 48 hours versus removal after discontinuation of the epidural catheter. In this study, the rate of re-catheterization was 12.4% higher in the early removal group, but there was no difference in the rate of urinary tract infections between the early versus delayed catheter removal groups. These authors concluded that removal of the urinary catheter should occur after discontinuation of the epidural catheter. Of note, neither of these studies considered patients with benign prostatic hyperplasia.

Similar to these studies, we also found an overall rate of catheter reinsertion of 10% in patients with an epidural catheter, and this was not statistically different than those without epidural. The presence of an epidural catheter did not result in a higher rate of UTI compared to those without one. This lack of statistical significance may be due to the limited number of patients who developed urinary tract infections. Patients with an epidural catheter had a longer duration of urinary catheters, which can be explained by our practice of leaving catheters in place until discontinuation of the epidural. Patients with an epidural catheter in place also had longer hospitalizations. This finding may be explained by the fact that early catheter removal may result in early patient mobilization which has been found to improve the postoperative course of patients after thoracic surgery [11] or selection bias by keeping urinary catheters longer after larger and more complex surgery.

The most significant finding in our study was the fact that patients with benign prostatic hyperplasia with an epidural catheter in place after a thoracic surgical operation have a three-fold rate of urinary retention when their urinary catheter is removed early, compared with patients without epidural anesthesia. It is interesting that there is no difference in the rate of urinary retention when all patients are looked as a whole, however when they are stratified by a specific modifier, BPH in this case, there is a striking difference. These findings may suggest that epidural catheters may not necessarily be the cause of urinary retention, but other risk factors such as benign prostatic hyperplasia.

Our findings are supported by a recent study by Young et al. in which they found BPH and esophagectomy to be risk factors for catheter reinsertion in patients with thoracic epidural anesthesia [8]. Our results suggest that urinary catheters should remain in place in patients with a history of BPH, at least until the epidural catheter has been discontinued. Our institutional practice has become to administer oral selective α_1_-adrenergic receptor antagonists for at least 24 hours prior to removal of urinary catheters, to reduce the risk of recatheterization.

### Limitations

This study has certain limitations. First, doses of other drugs that may impact urinary retention such as sympathomimetics and narcotics were not accounted for and may be affected by the epidural blockade. Second, our study lacks data regarding pain scores and the amount of rescue anesthetic use, which may be helpful in assessing the effectiveness and thus the successful positioning of the thoracic epidural catheter for each patient. Finally, our institutional practice has been to include local anesthetic (lidocaine) instead of narcotics in the thoracic epidurals, therefore, our results may not be generalizable to centers where the standard epidural infusion contains a different concentration or includes narcotics.

## Conclusion

Urinary catheters in patients with thoracic epidural analgesia can be safely removed in up to 90% of patients undergoing thoracic surgery, as evidenced by low reinsertion and infection rates. However, patients with a history of benign prostatic hyperplasia have a higher incidence of urinary recatheterization. In our cohort, 27% of these patients required urinary recatheterization as did 9% of males without benign prostatic hyperplasia.

Based on these findings, we recommend removal of urinary catheters in all women undergoing thoracic surgery, including those with epidurals within 24 hours of surgery, and males without benign prostatic hyperplasia, but they should be informed that their risk of reinsertion is approximately 10%. Urinary catheters should not be removed in patients with benign prostatic hyperplasia until 24-hour resumption of α_1_-adrenergic receptor antagonists to avoid unnecessary patient discomfort associated with recatheterization.

## Figures and Tables

**Table 1:** Characteristics of patients with and without epidural catheters undergoing thoracic surgery.

	Total Cases (n=267)		Epidural (n=88)		No Epidural (n=179)		p-value

**Male, n (%)**	124 (46)		46 (52)		78 (44)		0.18

**Age at surgery, years, median (range)**	66 (17 – 88)		68 (26 – 86)		65 (17 – 88)		0.04*

**Type of Surgery, n (%)**							

**Pulmonary Resections**	135 (51)		32 (36)		103 (56)		0.001*
**Esophageal Procedures**	43 (16)		21 (24)		22 (12)		0.02*
**Pleural Procedures**	39 (15)		19 (22)		20 (11)		0.02*
**Mediastinal Resections**	19 (7)		7 (8)		12 (7)		0.70
**Chest Wall Procedures**	19 (7)		8 (9)		11 (6)		0.38
**Hernia Repairs**	12 (4)		1 (1)		11 (6)		0.11

**Catheter Reinsertion, n (%)**	20 (7)	M: 14	9 (10)	M: 6	11 (6)	M: 8	0.23
		F: 6		F: 3		F: 3	

**Urinary Catheter Duration, days, median (range)**	1 (0 – 18)		4 (1 – 18)		1 (0 – 15)		<0.001*

**Postoperative Length of Stay, days, median (range)**	4 (0 – 85)		7 (2 – 85)		3 (0 – 44)		<0.001*

**Urinary Tract Infections, n (%)**	4 (1)	M: 1	3 (3)	M: 1	1 (1)	M: 0	0.10
		F: 3		F: 2		F: 1	

M=male, F=female;

* Statistically significant

**Table 2:** Characteristics and outcomes of patients with and without benign prostatic hyperplasia

	Total Cases (n=124)	BPH (n=15)	No BPH (n=109)	p-value

**Catheter Reinsertion, n (%)**	14 (11%)	4 (27%)	10 (9%)	0.05*
**Urinary Catheter duration, days, median (range)**	1 (0 – 18)	3 (1 – 13)	1 (0 – 18)	
**Epidural Catheter, n (%)**	46 (37)	8 (53)	38 (35)	0.16

BPH = benign prostatic hyperplasia;

* Statistically significant
